# Coping with stress in medical students: results of a randomized controlled trial using a mindfulness-based stress prevention training (MediMind) in Germany

**DOI:** 10.1186/s12909-016-0833-8

**Published:** 2016-12-28

**Authors:** S. M. Kuhlmann, M. Huss, A. Bürger, F. Hammerle

**Affiliations:** 1 Department for Child and Adolescent Psychiatry and Psychotherapy, University Medical Center of the Johannes Gutenberg University Mainz, Langenbeckstraße 1, 55131 Mainz, Germany; 2 Department University Hospital of Wuerzburg, Center of Mental Health, Department of Child and Adolescent Psychiatry, Psychosomatics and Psychotherapy, Fuechsleinstraße 15, 97080 Wuerzburg, Germany

**Keywords:** Medical students, Distress, Stress, Stress prevention, Mindfulness

## Abstract

**Background:**

High prevalence rates of psychological distress in medical training and later professional life indicate a need for prevention. Different types of intervention were shown to have good effects, but little is known about the relative efficacy of different types of stress management interventions, and methodological limitations have been reported. In order to overcome some of these limitations, the present study aimed at evaluating the effect of a specifically developed mindfulness-based stress prevention training for medical students (MediMind) on measures of distress, coping and psychological morbidity.

**Methods:**

We report on a prospective randomized controlled trial with three study conditions: experimental treatment (MediMind), standard treatment (Autogenic Training) and a control group without treatment. The sample consisted of medical or dental students in the second or eighth semester. They completed self-report questionnaires at baseline, after the training and at one year follow-up. Distress (Trier Inventory for the Assessment of Chronic Stress, TICS) was assessed as the primary outcome and coping (Brief COPE) as a co-primary outcome. Effects on the psychological morbidity (Brief Symptom Inventory, BSI) as a secondary outcome were expected one year after the trainings.

**Results:**

Initially, *N* = 183 students were randomly allocated to the study groups. At one year follow-up *N* = 80 could be included into the per-protocol analysis: MediMind (*n* =31), Autogenic Training (*n* = 32) and control group (*n* = 17). A selective drop-out for students who suffered more often from psychological symptoms was detected (*p* = .020). MANCOVA’s on TICS and Brief COPE revealed no significant interaction effects. On the BSI, a significant overall interaction effect became apparent (*p* = .002, η2partial = .382), but post hoc analyses were not significant. Means of the Global Severity Index (BSI) indicated that MediMind may contribute to a decrease in psychological morbidity.

**Conclusion:**

Due to the high and selective dropout rates, the results cannot be generalized and further research is necessary. Since the participation rate of the trainings was high, a need for further prevention programs is indicated. The study gives important suggestions on further implementation and evaluation of stress prevention in medical schools.

**Trial registration:**

This trial is recorded at German Clinical Trials Register under the number DRKS00005354 (08.11.2013).

## Background

The experience of distress during medical training is a well-known issue and much effort has been made to examine those effects on the students’ mental health and to initiate preventive programs [1]. Studies from Germany and other western countries revealed increased risks for the development of psychological disorders in populations of medical students. Depressive symptoms, for example, are reported to affect 8.8 to 58.2% of the student population [2–6]. The prevalence of burnout ranges between 17.2 and 71% [5, 7–9] and between 5.1 and 32% for anxiety disorders. To verify the assumption that medical training contributes to the deterioration of mental health, Brazeau et al. [10] compared matriculating medical students with age-matched college graduates from the general population. They found that medical students at the beginning of their studies were of better mental health compared to the controls, suggesting that the training process may contribute to an increase in psychological morbidity. These findings are confirmed by Kötter et al. [11] in an ongoing longitudinal study, which compared freshmen of medical training with students of other courses of studies in terms of mental health and also reported no differences at baseline assessment. However, there are no definite results demonstrating that medical students are basically more burdened by psychological morbidity during their studies than other high-achieving student populations. Henning et al. [12], for example, indicated that medical students have a similar quality of life in terms of physical and psychological health as non-medical students. In contrast, Aktekin et al. [13] showed a higher psychological distress in medical students compared to students studying economics and physical education.

Compared to the general population, there is a clear indication that burnout and low physical and psychological health is more prevalent among physicians at each stage of their career [5, 12]. In order to address the impending risk of developing mental health problems during training and in later professional life, health promotion and prevention programs have become prevalent in medical schools. The structure and content of interventions vary and include relaxation training, mindfulness-based stress reduction, mentoring programs, and stress management or self-hypnosis [14–16]. The use of mindfulness-based intervention programs has become very popular in medical schools and it has been proven to be an effective method, as it positively affects negative emotions, mental distress and perceived stress, and enhances self-efficacy, empathy and self-compassion [17–21]. The long-term effect of mindfulness courses, however, had only been evaluated by a few studies in a controlled condition [18, 22]. The positive effects on perceived stress or mental distress became apparent directly after the training but did not persist at follow-up. After six months an increase of self-compassion and self-efficacy remained. This demonstrates a need for further follow-up assessments to evaluate the preventive effects of mindfulness-based intervention. Additionally, it should be considered how to maintain the promising effects and if this could be reached more by adapting mindfulness intervention programs to the needs of medical students.

Although there are a wide variety of intervention programs that have been validated for use in medical training, little is known about their relative efficacy. Until now, only Jain [23] conducted a three-armed trial comparing mindfulness meditation, relaxation training, and a waitlist control group. Both training conditions showed comparatively good results in reducing mental distress and improving a positive state of mind. Furthermore, the study revealed a specific effect of mindfulness meditation in reducing distractive and ruminative thoughts. To confirm this unique mechanism, there is further need of a comparison of different approaches of stress prevention programs in medical school.

In a meta-analysis, Yusoff [1] has shown that stress prevention trainings are associated with moderate effects on medical students’ psychological health, and it has become evident that interventions of a duration of 4 to 8 weeks result in large effect sizes. Regarding the quality of available studies, systematic reviews [14, 15] report methodological limitations that should be addressed by future research. Forthcoming research should include the use of control groups, random allocation and long-term follow-up assessments. Furthermore, it should be considered to use a random sample of subjects instead of recruiting volunteers in order to minimize motivational effects [15]. As nothing is known at which stage of study a prevention training achieves the best health promoting effects, impacts of intervention on different stages of medical training should be compared [15]. Considering the fact that randomized controlled trials meet the requirements of a high scientific standard, the necessity of using a rigorous scientific method is called for.

Although programs are used to promote health in medical schools at several universities in Germany [24], to the best of our knowledge no randomized controlled trial of a prevention program for German medical schools has yet been published. In order to face the psychological health of medical students, our study aimed at evaluating the effect of a specifically developed mindfulness-based stress prevention training for medical students (MediMind; MM) on measures of distress, coping and psychological morbidity. To overcome some of the methodological limitations, we carried out a comparison with a well evaluated relaxation technique as a standard treatment of stress reduction (Autogenic Training; AT) and a control group (CG) in a three armed randomized controlled trial. This would enable us to compare the efficacy of two different kinds of intervention. Furthermore, we realized a follow-up assessment in all three study groups to measure long-term effects of stress prevention. Addressing potential discrepancies, we included students of different study sections.

MediMind has a special focus on the challenges and issues of medical training and provides concrete coping strategies. Therefore, we expected MediMind to have a more beneficial effect on the experience of stress, the use of functional coping strategies and psychological morbidity than the standard treatment and the control group.

## Methods

### Design

This was a prospective, three arm (experimental treatment, standard treatment and a control group without treatment), randomized, controlled trial with three assessment time points (baseline, post-intervention and follow-up one year post-intervention). This design was created in order to analyze both short term and long term effects while examining potential effects of the training in comparison to the waitlist control group and standard treatment with Autogenic Training.

According to the description in the study protocol [25], a sample size of 126 participants at the 5-year follow-up was calculated leading to an initial sample size of 189 participants. Related publications [20, 23, 26] used different designs and statistical approaches, with non-randomization or experimental vs. wait-list control group design. This lead to a synopsis, integration and analysis with effect sizes of the different outcome measures (F-values respective η-square-values, Cohen’s d [27] and Λ for multivariate approaches) with G*Power [28] and power calculation with 80% [29].

This trial received approval from the local ethics committee (Landesärztekammer Rheinland-Pfalz, file number: 837.380.13/9065-F) and the University Medical Center data protection official. All participants provided written informed consent according to the 1964 Helsinki declaration and its later amendments.

### Participants

The study was offered to medical students in the second and eighth semester of the Johannes Gutenberg University in Mainz (Germany). Because the curriculum and learning environment of dental students in the preclinical semesters is nearly analogous to that of medical students, dental students in their second semester were also included. The trainings (MediMind and Autogenic Training) were provided as a voluntary extra-curricular activity and students were introduced to the study design and the opportunity of participation at lectures and by written information. In order to secure privacy, students were informed that all health data was strictly secured and held separately from their medical faculty. The signed informed consent was a requirement to take part in the baseline assessment. At the post-intervention assessment time point, 50.- € vouchers were raffled among the participants as an acknowledgment of their participation. As a motivating incentive the participants of the control group received a 20.- € voucher. Because of the low response rate, everyone who participated in the follow-up assessment received a 20.- € voucher.

The recruitment started in November 2013. Unfortunately, due to study limitations, the recruitment had to be terminated before full sampling had been completed.

### Interventions and trainers

The training was parallelized in both treatment groups and each intervention was presented over a period of five weeks with weekly sessions of 90 min. This also included the provision of an accompanying booklet or handout to the participants containing the contents of each training session and the instructions for practice assignments. The treatment groups were led by a total of four trainers (clinical psychologists, licensed in psychotherapy with relevant experience in mindfulness interventions, and one physical education instructor certified as a trainer of Autogenic Training). Two of the trainers led MediMind and the other two trainers the Autogenic Training. There was no change between the trainers. The staff was instructed in the intervention trainings and followed a comprehensive operation manual. Participants of the control group remained without treatment but participated in the assessment time points. MediMind will be offered to them when the five-year follow-up assessment is completed.

A detailed description of the trainers’ qualifications as well as the contents and sequence of trainings (MediMind and Autogenic Training) is published in the study protocol [25].

### Mindfulness-based stress prevention training for medical students (MediMind)

In order to develop an intervention program tailored to the needs of students in medical education, interviews with the target group were conducted beforehand. Students often mentioned the need to acquire specific action-oriented strategies to help them face stressful situations, such as examinations or high workload. This was taken into consideration when developing MediMind and combining mindfulness aspects with approaches from cognitive behavioral therapies. This implies learning how to relate to one’s own experiences in a more accepting and non-judgmental way, and helps one not to be overwhelmed by thoughts and feelings. Therefore, mindfulness meditation was practiced in each training session and CDs were provided for home practice. The participants were introduced to the ‘satellite-position’ as a target state of mindfulness and learned how to observe thoughts, emotions, physical reactions and impulse to act. In exercises, they became familiar with the presence-of-mind attitude in order to realize and target stress constructively. In this context, participants learned to address intrusive and distracting thoughts and feelings in an accepting attitude in order to feel less involved and to reduce their stressful impact. Another concept known to be effective in preventing stress is represented by the approaches of cognitive behavioral therapy [30]. This focuses on the stress heightening influence of dysfunctional cognitive judgment mechanisms and follows the approach of change and action-oriented strategies that do not require long-term training and work as skills in dealing with stressful situations. In this context, the students became aware of the impact of cognitive judgment mechanisms and core beliefs (‘I always have to perform perfectly!’) on our feelings and level of stress. This was taken into account by additionally implementing stress-management techniques of the cognitive behavioral therapies to our training. The participants learned how to detect dysfunctional cognitive judgment mechanisms (errors in reasoning) and practice the use of functional reevaluation. Additionally, the stress heightening influence of personal standards and assumptions were discussed. The students were introduced to various experiments in order to find a health-promoting way of dealing with these personal standards and assumptions. To cope with tense situations, the use of stress-tolerance skills and the concept of radical acceptance was imparted [31].

The combination of acceptance strategies (concept of mindfulness) with change strategies (contents of cognitive behavioral therapies) enabled the participants to be less reactive when experiencing stress and to decide more deliberately whether change is possible. This offered the possibility to either modify the situation, adapt their judgment mechanisms, or to otherwise meet these conditions with acceptance. This combination follows the concept of dialectical behavior therapy developed by Linehan [31]. The extension of mindfulness involving strategies to change dysfunctional cognitive assessment-mechanisms is also described in Lehrhaupt & Meibert [32] and Hassed et al. [33]. Practice assignments helped to generalize the effects of the training and to apply the techniques in everyday situations.

### Autogenic training

The basic skills of Autogenic Training according to the Schulz method [34] were practiced in this intervention group. As it is an auto-suggestive relaxation technique, the participants learned how to instruct themselves to suggest specific autonomic sensations such as muscular relaxation, vascular dilatation, stabilization of heart function or regulation of breathing [35]. These instructions consist of six exercises with corresponding formulas that are subvocally repeated (e.g. ‘My arm is very heavy’). Additionally, the training is extended by exercises including progressive muscle relaxation, breathing relaxation, exercises for body awareness, imaginary journeys and qigong movements. Individual practice outside the training was supported by informational material.

### Measures

In the present study, we report the results of three assessment time points: (1) baseline, after receiving signed informed consent and before random assignment to the study groups; (2) post intervention, three weeks after the last training session; (3) follow-up, one year post trial. At each assessment time point, data of the primary, co-primary and secondary outcome measures were collected. Standard demographic measures were assessed at baseline. Additionally, at post intervention and follow-up assessment participants were asked to assess how often per week they used the strategies that were taught in the trainings.

### Primary outcome measure

Trier Inventory for the Assessment of Chronic Stress (TICS) [36]. The TICS consists of 57 items to evaluate different aspects of chronic stress by assessing nine subscales: ‘work overload’, ‘social overload’, ‘excessive demands from work’, ‘lack of social recognition’, ‘work discontent’, ‘social tension’, ‘pressure to perform’, ‘social isolation’ and ‘chronic worrying’. The answers are rated on a Likert-type scale ranging from 0 (‘I never experienced this’) to 4 (‘I experienced this very often’). In order to assess the experience of chronic stress, an additional Chronic Stress Screening Scale (SSCS) can be computed that consists of 12 items taken from the other dimensions (‘chronic worrying’, ‘work overload’, ‘social overload’, ‘excessive demands from work’ and ‘lack of social recognition’). Cronbach’s α ranged from .84 to .91 and is classified as good to excellent. Validity was confirmed by a principal component analysis resulting in a nine-factor solution and plausible correlations between the TICS and other stress questionnaires [36].

### Co-primary outcome measure

Brief COPE [37]. The Brief COPE assesses 14 dimensions of effective and ineffective coping strategies: ‘self-distraction’, ‘active coping’, ‘denial’, ‘substance use’, ‘use of emotional support’, ‘use of instrumental support’, ‘behavioral disengagement’, ‘venting’, ‘positive reframing’, ‘planning’, ‘humor’, ‘acceptance’, ‘religion’ and ‘self-blame’. It consists of 28 items with four response categories of a Likert-type scale ranging from 1 (‘not at all’) to 4 (‘very much’). Cronbach’s α range from .50 to .90 and reflects poor to good internal consistency. Construct validity could be confirmed by a nine-factor solution similar to the full inventory [37]. Convergent and discriminant validity is confirmed by plausible correlations between coping strategies and personality qualities [38]. The Brief COPE is available in a German translation and has been validated [39].

### Secondary outcome measure

Brief Symptom Inventory (BSI) [40]. The BSI measures psychological distress based on nine subscales: ‘Obsessive-Compulsive’, ‘Paranoid Ideation’, ‘Hostility’, ‘Somatization’, ‘Depression’, ‘Interpersonal Sensitivity’, ‘Anxiety’, ‘Psychoticism’ and ‘Phobic Anxiety’. It consists of 53 items to be answered on a Likert-type scale ranging from 0 (‘not at all’) to 4 (‘extremely’). A global measure of overall psychological distress is provided as an average response on each item (Global Severity Index; GSI). A German version is available and internal consistency coefficients (Cronbach’s α) show good results ranging from .70 to .88 in a community sample. Validity is proven by plausible correlations between the BSI and other instruments [41].

### Randomization process

After signing informed consent and baseline evaluation, participants were randomized to one of the three study groups (MediMind, Autogenic Training or control group) [25]. In order to control for potential confounding effects, students were stratified randomized by course of study (medical versus dental), semester (2nd or 8th) and sex. Regarding maximum power for analysis between experimental versus standard treatment, an allocation ratio with 2 (MediMind) : 2 (Autogenic Training) : 1 (control group) was realized. Randomization was operationalized via drawing lots by an independent member of the institute not involved in the project.

### Statistical analysis

All analyses (except sample size calculation as stated above) were conducted using IBM SPSS Statistics, Version 23. A *p*-value of < .05 with an α-level of 5% set for statistical significance. Following a conservative procedure, reducing potential α-errors when imputing data [42], the two-folded approach [43] stated in the study protocol was reduced to a completer-analysis including all persons with complete data-sets.

Descriptive data at baseline was further analyzed with *t*-tests for metric variables and X^2^-tests for binary variables, respective Fisher’s Exact Test if expected cell frequency was less than five [42]. Differential analysis with *t*-tests of missing data, response, drop-out rates and time of post-data collection were used to access potentially confounding effects.

All metric variables were tested for normality via Kolmogorov-Smirnoff-Tests [44] resulting in non-normality in the secondary outcome and normalization via logarithmic transformation of the data [45].

In order to reduce multiple testing with familywise Type I errors [46] for primary, co-primary and secondary outcome, MANCOVA’s with group x time interactions and the covariates gender and time of post-data collection (due to differences stated below) were performed. Post hoc MANCOVA’s were applied with Bonferroni-Holm [47] corrections for multiple testing regarding differential interactions between MediMind and control group and MediMind and Autogenic Training. Separate scales of the instruments for the outcomes were tested in post hoc repeated measures ANCOVA’s with Bonferroni-Holm correction.

Partial η-square values according to Cohen [27] were calculated for estimating effect sizes referring to interaction effects with cut-off-norms of η-square values ≥ .0099 denoting to a small effect size, η-square values ≥ .0588 denoting to a medium effect size and η-square values ≥ .1379 pointing to a large effect size.

## Results

### Baseline characteristics

**Fig. 1:**
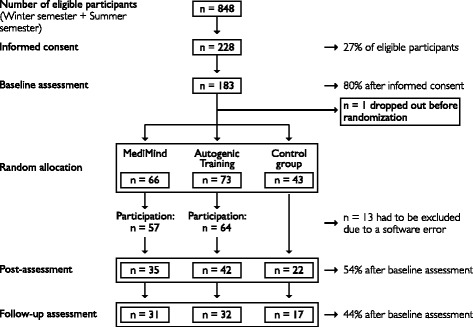
Flow of participants

Data of 80 participants were included for the evaluation. Figure 1 shows the flowchart of participants in the different study stages. A total of 228 signed informed consent, 80% (*n* = 183) completed baseline assessment and of this sample 61% (*n* = 112) returned post-measures. Due to an error in the online version of the baseline assessment, 13 participants had to be excluded from the evaluation (MM: *n* = 6, AT: *n* = 6, CG: *n* = 1). One participant dropped out before allocation to the study groups. At one year post-intervention, a sample of 80 students with complete data-sets could be included in the per-protocol analysis. Overall, seven groups of MediMind and seven groups of Autogenic Training were conducted each comprised of six to eleven participants.

The characteristics of the study groups for the per-protocol analysis are summarized in Table 1. There were no statistically significant differences in age or for study stage (preclinical and clinical cohort) between the groups; however, there was no equal distribution regarding sex. Some participants did not return post and follow-up measurements directly after they were asked to complete the questionnaires. An analysis was run to examine in how the groups differed with regard to questionnaires return rate. Analysis of Variance (ANOVA) revealed a significant difference between the groups (*F* (2,77) = 16.870, *p* = .000) for the time of post-data collection. The control group returned the questionnaires on average 2.5 weeks later than the two other groups (CG: *M* = 4.5, *SD* = 1.8; MM: *M* = 2.5, *SD* = 1.3; AT: *M* = 2.3 *SD* = 1.3). The time of the receipt of the one-year follow-up measurements did not differ between the groups.

**Table 1:** Characteristics of participants by study group at baseline

	Total *N* = 80	MediMind *n* = 31	Autogenic Training *n* = 32	Control group *n* = 17	MM vs. AT *p-value*	MM vs. CG *p-value*	AT vs. CG *p-value*
Mean age (SD) in years	23.39 (3.91)	23.29 (2.81)	23.72 (5.12)	22.94 (3.09)	n.s.^a^	n.s.^a^	n.s.^a^
Female sex, N (%)	67 (84%)	30 (97%)	27 (84%)	10 (59%)	.003^b^		
Preclinical cohort, N (%)	48 (60%)	16 (52%)	22 (69%)	10 (59%)	n.s.^b^		
Clinical cohort, N (%)	32 (40%)	15 (48%)	10 (31%)	7 (41%)			

^a^ *t*-test, ^b^chi-squared test or Fisher’s Exact Test

Further analysis of overall baseline assessment (*n* = 158) revealed that the level of distress was high. Compared to a reference sample of the general population, which is provided by the authors of the TICS [36], 39.9% of the medical students scored above a T-score of 60 for the Chronic Stress Screening Scale (SSCS) of the TICS, whereas consistently with the normal distribution 15.87% were to be expected [48]. A student sample as reference was not available. With regard to psychological morbidity, medical students of our study showed higher psychological morbidity at baseline than a student population given as a reference sample by the authors of the BSI [41]. Of the students, 18.4% scored above cut-off (T score > 63) on the Global Severity Index (GSI) as a measure of overall psychological distress. With regard to the T-distribution, it exceeded the expected percentage of 9.68 by the factor 2 [48].

To explore differences within the medical training, we compared the preclinical and clinical cohort of our sample. The level of overall psychological distress (GSI) in the preclinical cohort was significantly higher at baseline than in the clinical cohort (t (155) = 2.216; *p* = .028; preclinic: *M* = .70, *SD* = .51; clinic: *M* = .52, *SD* = .40). No differences could be found for the amount of perceived stress (SSCS).

**Fig. 2:**
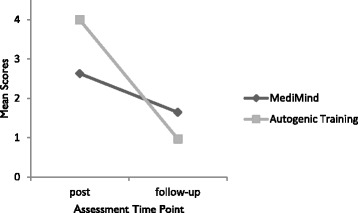
Means of strategy use frequency

Concerning the number of training units in which the participants took part, there was no significant difference between the two training groups (Table 2). Additionally, it was analyzed whether the participants of MediMind or Autogenic Training differ with regard to strategy use frequency. A repeated measures ANOVA revealed a significant interaction effect between the groups (*F* (1,61) = 8.749, *p* = .004, *η* ^*2*^ = 0,125). According to this, students who received Autogenic Training practiced more often directly after they finished the training (post-intervention), but had a stronger reduction in frequency of practice (follow-up) than students who received the MediMind training (Fig. 2).

**Table 2:** Frequency of participation by study group

Study Group	Perceived training units				
	1	2	3	4	5
MediMind (*n* = 31)	0%	0%	3%	29%	68%
Autogenic Training (*n* = 32)	0%	0%	6%	22%	72%

### Analysis of dropout rates

An analysis was conducted to determine if there was a systematic dropout effect. Those participants who completed every assessment (per-protocol participants) were compared to those who dropped out after the baseline assessment (non-starters). A *t*-test of the Global Severity Index (BSI) revealed significant differences (*t* (107.452) = -2.365, *p* = .020). Students who dropped out of the study had a significantly higher psychological morbidity (GSI: *M* = .77, *SD* = .57) at baseline assessment than students who completed the study and constituted the per-protocol sample (GSI: *M* = .56, *SD* = .40). The dropout rates of the preclinical and clinical semester did not differ. Aside from the missing assessments, the participation rate in the trainings was higher. Figure 1 shows that participation in the training was higher than the response rate of the questionnaires. The evaluation of data therefore, does not include every student who attended the training.

### Primary and co-primary outcome

A repeated measures multivariate analysis of covariance (MANCOVA) was run for the primary outcome in order to compare the experimental treatment (MediMind), standard treatment (Autogenic Training) and control group. The scores of the three assessment time points were entered as dependent variables and the group status as the independent variable. Sex and time of post-data collection were entered as covariates in order to take the significant group differences into account.

No significant interaction effect (group x time) for the TICS can be stated (*p* = .502, *η* ^2^ = .234). In order to examine interaction effects of the SSCS (Chronic Stress Screening Scale), repeated measures analysis of covariance (ANCOVA) with sex and time of post-data collection as covariates was conducted and revealed no significant result (*p* = .251, *η* ^2^ = .035). The longitudinal changes for each group across time points are presented in Table 3.

**Table 3:** Outcomes on Chronic Stress Screening Scale (SSCS) and Global Severity Index (GSI)

	MediMind (*n* = 31)	Autogenic Training (*n* = 32)	Control group (*n* = 17)
Chronic Stress Screening Scale (SSCS)			
pre	21.29 ± 8.86	19.44 ± 7.93	22.18 ± 7.49
post	18.58 ± 8.09	18.81 ± 8.15	20.35 ± 8.94
follow-up	20.42 ± 7.81	22.72 ± 8.79	19.47 ± 7.16
Global Severity Index (GSI)			
pre	.55 ± .44	.57 ± .37	.56 ± .39
post	.54 ± .52	.58 ± .44	.49 ± .40
follow-up	.43 ± .34	.66 ± .54	.55 ± .41

*Note.* Means ± SD

To examine differences in co-primary outcome, a repeated measures MANCOVA was run with sex and time of post-data collection as covariates. The scores of the three assessment time points were entered as dependent variables and the group status as the independent variable. There is no significant interaction effect (group x time) for the Brief COPE across the three assessment time points (*p* = .237, *η* ^2^ = .408).

### Secondary outcome

**Fig. 3:**
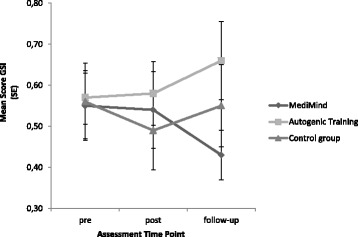
Means of Global Severity Index (GSI)

At the time of the one-year follow up an impact on psychological morbidity was assumed. Statistical analysis of the secondary outcome (BSI) was run with a repeated measures MANCOVA entering the nine scores of the BSI as dependent variables, with the group status as the independent variable and the covariates sex and time of the post-data collection. A significant interaction effect (group x time) was found (*F* (36,118) = 2.027, *p* = .002, *η* ^2^ = .382), which indicates that the three groups differ in psychological morbidity over time. Post hoc MANCOVA’s with Bonferroni-Holm correction (α-level of 2,5%) regarding differential interactions between MediMind and the control group revealed a significant interaction effect (*F* (18,27) = 3.239, *p* = .003, *η* ^2^ = .683) but not between MediMind and Autogenic Training (*p* = .087, *η* ^2^ = .416). Further post hoc tests that protect against Type I error revealed no information concerning the type of difference on the subscales between the groups. The plot of means (Fig. 3) of the Global Severity Index (GSI) as a measure of overall psychological distress helps to provide information on the direction of change. The descriptive information of longitudinal changes for each group across time points is presented in Table 3. Although there is no significant difference of the GSI between the groups, the plot of means demonstrates a change in the predicted direction as MediMind showed a reduction of psychological distress one year after the training. In contrast to this, Autogenic Training and the control group seem to have deteriorated with respect to psychological distress.

## Discussion and conclusion

In terms of the primary and co-primary outcome, our study revealed no significant interaction effect. Therefore, a positive effect of the interventions on the experience of stress or the use of functional coping strategies could not be proved. As previously assumed, an effect on psychological morbidity could be noticed one year post trial. Statistical analysis revealed a significant change on the subscales of the BSI between MediMind and the control group, but post hoc tests prevent any further interpretation of results. Considering the means of the Global Severity Index (GSI), students who participated in MediMind suffered less from psychological symptoms one year after the training compared to participants of the Autogenic Training and the control group. Due to the lack of significant post hoc tests, this observation does not statistically confirm a preventive efficacy of MediMind. The increase of distress in the other study groups (Autogenic Training and control group) could be due to the imminence of the preliminary exams in medical education and the final exams in medical education after one year. Nevertheless, it can be noticed that even one year later, participants still used the strategies they had learned in the trainings. The frequency of using the skills that were taught in MediMind did not decrease as much as it did in the Autogenic Training. This result may indicate that the skills of MediMind are more practical and compatible with the daily realities of medical students and that they require less effort in practicing.

Our study did not succeed in confirming the promising results of the three-armed randomized controlled trial conducted by Jain [23]. They provided evidence for health promoting effects of stress prevention trainings in medical schools and revealed mindfulness meditation to be unique in reducing ruminative and distractive thoughts compared to a relaxation training. It might be suggested that mindfulness meditation may provide a unique mechanism in reducing mental distress. In view of our results, differential effects of the concept of mindfulness and somatic relaxation were not apparent, as there were no significant differences in the use of coping strategies. Since no further study exists that compares different approaches, no conclusion can yet be drawn about their relative efficacy and which type of training works best for whom. To assess the efficacy of mindfulness, studies of different standards are available. With reference to results of two-armed randomized controlled trials, mindfulness meditation shows good effects in reducing distress or enhancing mental health of medical students [18–20, 22], whereas one study investigating mindfulness meditation could not confirm these effects [49]. In comparison, MediMind is unique in its combination of mindfulness meditation and teaching skills that enable the student to reduce a stressful experience when change is possible. This should have the advantage to provide the participants with specific strategies that can be used directly without the necessity of long-term practice. A loss of motivation as a probable long-term effect could thereby be reduced.

In order to optimize control of possible confounding factors we conducted a randomized controlled trial with a comparison of three study groups that made it possible to examine two different types of interventions and to realize a follow-up assessment. In contrast to other studies [20, 23, 50, 51], we decided on a conservative approach by conducting a per-protocol analysis, correcting for multiple testing and interpreting the interaction effect of the repeated measures MANCOVA as an evidence of the effectiveness of the trainings. Since stress prevention programs seem to affect the experience of distress and mental health of medical students in other countries positively, the question is how the results of our study can be explained. In this context the limiting effect of the high dropout rate has to be discussed, but the response rate and the participation rate should be treated separately. As it can be seen in Fig. 1, the participation rate of the trainings was higher than the return of post-measurements. For MediMind and Autogenic Training, the participation was nearly twice as high as data was available. This shows a high motivation for attending the trainings, but not for filling in the questionnaires. Since post assessment time points overlap with the exam period, this may explain the high dropout rate. Furthermore, regarding the control group, the offer of a participation in the trainings five years after the start of our study might have caused in a decrease of motivation. This may explain that the highest dropout rate in our study was found for the control group. It has to be questioned if the control group represents the natural progress of data and is therefore suitable as a control condition. In terms of the response rate it has to be stated, that after baseline assessment nearly 50% of the students did not return post-measurements. A comparable dropout rate is also reported by McGrady et al. [50], who only had a response rate of 49.2% at post-assessment. Statistical analysis at baseline revealed that students who dropped out of our study were of significantly poorer mental health compared to the students who formed our evaluation sample, and it can be assumed that this had an impact on our results. It is well known that approaches in primary prevention of mental disorders face small effects, thus a reduction in sample size might impede results in a way that small effects cannot be detected [52, 53]. Furthermore, it is recognized that the highest effect of an intervention arises in participants who are initially heavily burdened [54]. Therefore, students who would possibly have improved the most by the trainings are not included in our evaluation. This assumption is supported by McGrady et al. [50], who found consistently lower depression scores in a high risk group after the intervention, compared to low risk students. Therefore, our data do not provide a reliable statement on every participant and do not allow drawing conclusions on the effectiveness of stress prevention trainings in German medical schools.

Another limitation was the small sample size that involved a certain risk of not having enough power to detect potential effects. Due to study limitations, the recruitment had to be terminated before the required sample size had been reached. As we used an ambitious study design with three study groups, an inclusion of more participants than in other randomized controlled trials was required. To our knowledge, only Jain et al. [23] reported the effects of stress prevention in medical school by comparing three groups, whereas other studies chose a comparison of two groups or did not carry out a controlled condition. In contrast to a control group, Jain et al. [23] confirmed good effects for two different interventions (meditation and relaxation training) on perceived distress and positive mood. Furthermore, they detected differences between the two interventions concerning their mechanisms of action. In this approach they included a sample size which is similar to ours, but eligible participants had to “self-identify as currently experiencing a significant amount of stress” (*p*. 12) [23]. Therefore, it can be assumed that this pre-selection may have had an impact on the results since the participants were of poorer mental health at baseline. In terms of the Global Severity Index (GSI) assessed by the Brief Symptom Inventory, they included students with higher mental distress (*M* = .66, *SD* = .45) compared to our sample (*M* = .56, *SD* = .40).

With respect to our study design, it has to be mentioned that our data included students of preclinical and clinical semesters. Since these two cohorts differed significantly at baseline in terms of their overall psychological distress, this might moderate the overall effect of our study. Former studies on stress prevention trainings in medical school primarily included students in their first or second year of study [15], and therefore, these differences were of no consequence. It is still unclear if the positive results of previous approaches can be transferred to students at a stage of advanced studies. Our aim of comparing the effectiveness of stress prevention at different stages of studies could not be achieved because of the insufficient sample size. Furthermore, our sample has no equal distribution of sex which may lead to distortions and effect the generalizability. This limitation is in line with other studies [18, 23] that included predominantly female participants. With respect to the gender ratio in German medical schools, it should be stated that there is no equal distribution in sex and that female students predominate [55].

Future research on stress prevention in German medical schools should focus on how to improve the response rate. A successful approach seems to be offering a training as an elective course [20] or as a curricular tool [22] to minimize motivational effects. Since this was not possible for the realization of our trainings, we had to provide the training sessions as an extracurricular activity. In order to increase the response rate of the follow-up assessment, every participant who returned the questionnaire was rewarded by a voucher. This showed good effects and highlights the need of an external motivation in this field of research. It needs to be emphasized that this reward only had an effect on the response rate, but that no reward was necessary to increase the participation in the trainings, since the participation rate of the trainings was higher than the return rate of post-measurements. In conclusion, the present study provides important suggestions for future research. It will be necessary to improve the outcome of a randomized controlled trial by offering an appropriate and sensible implementation of a training in the curriculum. Time of post-measurements should be reconsidered to reduce the drop-out rate during exam periods. Moreover, incentives should be provided in order to increase the response rate, and finally, a waitlist control condition should be chosen.

## Conclusion

In accordance with former studies [2, 3, 7], our data confirm the high experience of stress and the vulnerability for psychological disorders in students of German medical schools. Since this is still evident in later professional life [5, 56], appropriate preventive interventions should already be provided during the time of studies to decrease the risk of suffering from mental disorders and to guarantee a stable performance of future doctors in patient care. Currently, no data are available on the effectiveness of stress prevention programs for students in German medical schools. Therefore, nothing is yet known about how an offer of a preventive training would be accepted by the students and how it could be successfully implemented in their curriculum. Our data demonstrate that the stress prevention training was well accepted by the participants and provided skills that were still used one year later. Due to the small sample size, the before mentioned objectives could not be answered conclusively. Although means of the Global Severity Index (BSI) indicate that MediMind may contribute to decreased psychological morbidity of the participants, a significant preventive effect cannot be proved. Due to the high dropout rate and the small sample size, the results cannot be generalized and can, therefore, not be regarded as conclusive. Future research is necessary to evaluate preventive programs in German medical schools and to determine how they can be implemented within the heavy course load in order to reach those students who are severely burdened. Moreover, the evaluation of a larger sample size is necessary to represent the gender ratio in German medical schools for differential analysis.

In conclusion, future research seems to be promising as the stress prevention trainings in our study were highly accepted, and with respect to preventing psychological disorders in future doctors, there is a need for action. To prevent a high dropout rate and to generate a sufficiently large sample, the design to assess post-intervention and follow-up data has to be optimized.

## Abbreviations

ANCOVA: Analysis of covariance
AT: Autogenic training
BSI: Brief symptom inventory
CG: Control group
GSI: Global severity index (BSI)
M: Mean
MANCOVA: Multivariate analysis of covariance
MediMind: Mindfulness-based stress prevention training for medical students
MM: MediMind - mindfulness-based stress prevention training for medical students
SD: Standard deviation
SE: Standard error
SSCS: Chronic stress screening scale (TICS)
TICS: Trier inventory for the assessment of chronic stress
